# Hack your organizational innovation: literature review and integrative model for running hackathons

**DOI:** 10.1186/s13731-023-00269-0

**Published:** 2023-03-03

**Authors:** Ben Heller, Atar Amir, Roy Waxman, Yossi Maaravi

**Affiliations:** 1 Baruch Ivcher School of Psychology, Reichman University (IDC), Herzliya, Israel; 2 The Adelson School of Entrepreneurship, Reichman University (IDC), Herzliya, Israel

**Keywords:** Creativity, Innovation, Hackathon, Organizational Innovation, Ideation

## Abstract

This article aims to offer a comprehensive overview of the existing literature on the hackathon phenomenon to offer scholars a common ground for future research and managers and practitioners research-based guidelines on best planning and running a hackathon. A review of the most relevant literature on hackathons was conducted to serve as the research basis for our integrative model and guidelines. This article synthesizes the research on hackathons to offer comprehensible guidelines for practitioners while also providing questions for future hackathon researchers. We differentiate between the different design characteristics of hackathons while noting their advantages and disadvantages, discuss tools and methodologies for successful hackathon setup and execution step-by-step, and provide recommendations to encourage project continuity.

## Introduction

### The importance of Organizational Innovation

“The riskiest thing we can do is just maintain the status quo”—Bob Eiger, CEO of Walt Disney (Iger, 2019).

This quote underscores the crucial importance of organizational innovation (Maradana et al., 2017). To remain relevant amidst the growing market competition, businesses, organizations, and even individuals (Linton & Klinton, 2019) must be innovative and entrepreneurial: frequently provide innovative products and services which give significant value to the customers and meet market demands (Laforet, 2011; Nolte et al., 2018). Today, innovation is ubiquitous, and some organizations even include it as an integral part of their vision and purpose of existence (Kahn, 2018). The definition of the term innovation varies (Maaravi et al., 2020), but a common one is “…production or adoption, assimilation and utilization of value-added innovation in economic and social fields. Innovation and expansion of products, services and markets; New methods of manufacturing and setting up new management systems. This is a process and a result.” (Crossan & Apaydin, 2010; p. 1155).

Several methodologies have been created to promote organizational creativity and innovation. One example is crowdsourcing, which allows organizations to involve the external environment—customers, suppliers, academics, and more—in creating ideas and making decisions (Flores et al., 2018; Herala et al., 2019). Another example is managing an active product/service-user community to involve them in the internal ideation and development processes and consult them about their needs (King & Lakhani, 2013).

The hackathon phenomenon—a portmanteau of the words “hack” and “marathon”—which are the focus of this article, represents a different methodology. Research shows that organizations often run hackathons to enhance innovation processes, both directly through ideation or prototyping of new products or indirectly by increasing employees’ motivation for innovation (Herala et al., 2019).

### Hackathons—a tool for promoting organizational innovation

The close connection between hackathons and the production of innovative ideas has been supported in the academic literature (e.g., Calco & Veeck, 2015; Maaravi, 2020). Hackathons are short-term and intense events, ranging from small and improvised to huge ones with many participants. At these events, diverse groups gather to solve a defined problem or create a joint project that meets a specific need determined by the event organizer. The method varies, but it usually entails competition or collaboration between teams for a common goal (Nolte et al., 2020).

Traditionally, hackathon groups consisted solely of programmers who needed to solve a technological problem or produce a prototype (Briscoe, 2014; Mishra & Tripathi, 2020), but this has changed. Many organizations have used hackathons to address diverse challenges and needs (Kolog et al., 2016). These include higher education (Gama et al., 2018; Kienzler & Fontanesi, 2017), research (Maaravi, 2018), science and medicine (Wang et al., 2018a), and internet communities (Cameron Craddock et al., 2016). Other domains include civic and government fields (Hartmann et al., 2019; Henderson, 2015), choreography (Briscoe & Hon, 2016), acoustics (De Winne et al., 2020), sustainability (Zapico et al., 2013), films and art (Valjamae et al., 2017) and more.

Examples of prominent companies which conduct yearly international hackathon events with tens of thousands of participants are Microsoft, Google, and Facebook, who host these events to allow employees to take time off their day-to-day job and team up to promote a project of their choosing (Pe-Than et al., 2018). Interestingly, the Facebook “Like” button was invented as part of the internal organizational hackathon, revolutionizing social networking (Briscoe, 2014).

Hackathons benefit both the organizers and the participants: by strengthening and developing employees by breaking routines, encouraging creativity, and increasing employees’ skillset (Chandrasekaran et al., 2018; Nolte et al., 2018). Other positive effects are networking between employees and strengthening their connection with the organization (Cobham et al., 2017; Fattah et al., 2021; Herala et al., 2019). Finally, organizations benefit from the acceleration and initiation of novel processes, the creation of a large pool of ideas, the identification of new talents, the opportunity to integrate external knowledge, and the contribution to the organization’s public image.

### Objective of the current article

This article reviews existing literature and offers scholars, managers, and innovation leaders an integrative model of running hackathons (Soleas, 2020). The review encompasses dozens of studies examining different hackathons using empirical research methodologies, varied statistical samples, and qualitative interviews with organizers and participants. Based on these studies, we classify and describe hackathons based on their focus (tech vs. issue), design characteristics (i.e., selective vs. open, competitive vs. collaborative, physical vs. virtual vs. hybrid), and discuss the distinct advantages and disadvantages of each. Subsequently, we rely on the literature to offer step-by-step guidelines on how to execute a hackathon best: starting from the necessary preparations needed before the event (e.g., defining the objective, team building, etc.), followed by the crucial innovation-driving steps during the event (e.g., ideation, judgment), and finally exploring the post-event factors which most contribute to project continuity. This article strives to contribute to scholars by mapping and integrating existing research, emphasizing progress, and identifying lacunas. Managers and innovation leaders will benefit from the practical, research-based tools and guidelines we offer for planning and running successful hackathon events.

## Related works

This article is among numerous articles, each with its methodology and analyses, offering practitioners and researchers practical guidelines on best executing hackathons based on a literature review. For example, Olesen and Halskov (2020) conducted an extensive literature review of hackathon articles published over ten years, discussed the state of hackathons research, and explored the motivations for using hackathons as part of research efforts. Jaakola et al. (2021) offer step-by-step chronological guidelines on executing short-term innovation events (e.g., hackathons, workshops) based on events that occurred as part of the BatlicSatApps project. Valença et al. (2020) focused specifically on corporate hackathons and systematically reviewed the relevant literature. Komssi et al. (2014) describe hackathons, their objectives, characteristics, and challenges, and give five examples of these factors. Finally, Pe-Than et al. (2018) detailed the design characteristics of hackathons and provided practical managerially-oriented guidelines on their basis following a minor review of the literature.

Nevertheless, none of these articles provide a holistic, systematic, research- and practice-oriented chronological model of hackathon execution best practices. Specifically, they either: (1) do not focus specifically on hackathons (Jaakola et al., 2021); (2) focus on one specific type of hackathon rather than hackathons in general (Valença et al., 2020); (3) are not systematic reviews or do not include an exhaustive mapping of the literature (Komssi et al., 2014; Pe-Than et al., 2018; Valença et al., 2020); or do not offer comprehensive, step-by-step guidelines on executing hackathons (Olesen & Halskov, 2020). These examples do not exhaust the literature of related works, as more reviews and hackathon guidelines exist. However, they are distinct enough and cover the necessary ground to exemplify this article’s unique position and contribution to the literature. See Table 1 in the “Conclusions” section for a concise summary of the related works and their relation to the current article.

**Table 1:** Comparison between the current article and related works

Articles	The current article (2022)	Komssi et al. (2014)	Pe-Than et al. (2018)	Olesen and Halskov (2020)	Valença et al. (2020)	Jaakola et al. (2021)
Criteria						
Focus	State-of-the-art review on planning and executing hackathons in a step-by-step fashion	Non-systematic review of hackathons in the technology and software domains	Non-systematic review on how to design hackathons in light of specific goals	Review of the hackathons’ usage in research	A systematic review focusing on corporate hackathons in the IT industry	Case study on how to execute short-term events in the tech and software domain in a step-by-step fashion
Methodology	A systematic review of the literature	Non-systematic review of the literature and case studies	Non-systematic review of the literature and case studies	A systematic review of the literature	A systematic review of the literature	Case study
Does it offer a process model?		x		x		
Stages of the process model	Divided into pre-, during-, and post-event guidelines, emphasizing project continuity	N/A	Divided into design choices and strategies based on goals	N/A	Divided into pre-, during-, and post-event guidelines	Divided into pre-, during-, and post-event guidelines
Main Contribution	Offers an in-depth, research- and practitioner-oriented, step-by-step model on how to best execute hackathons (regardless of domain or subject) and secure their success and continuation based on empirical literature	Details how hackathons are commonly used in the technology domains and describes such hackathons, their challenges, and outcomes	Describes hackathon participants’ and stakeholders’ organizational and personal goals and provides design guidelines on how to best achieve them	Maps how hackathons are used as both tools for research and subjects of research based on empirical literature	Focuses on corporate hackathons and offers a step-by-step model on how to execute them and secure their success based on empirical literature	Provides and tests a general step-by-step model on best executing short-term events (of which hackathons are one type)

## Review methodology

Our literature review methodology echoes Kraus et al.’s (2020) systematic literature review guidelines. Systematic literature reviews are both methodologically transparent and reproducible, with defined search criteria and high levels of objectivity. This approach contrasts with traditional literature reviews, which are opaque (i.e., the inclusion/exclusion criteria are not well-defined, databases are not stated, etc.), subjective, and may fall prey to personal bias. The most important steps when performing a systematic literature review are: (1) formulating research questions; (2) developing a review protocol; (3) identifying the studies (inclusion/exclusion criteria); and (4) extracting and synthesizing the data based on topics, not authors (Kraus et al., 2020).

### Research questions

This review attempts to answer the following questions: (1) What are the different design characteristics of hackathons? (2) What guidelines can we extract from the literature to create a practitioner-oriented integrative hackathon model? (3) Based on 1 and 2, what are the lacunas and gaps in the hackathon literature that future research should investigate?

### Inclusion/exclusion criteria

- Subject: As this review focuses on hackathons, we included articles that had displayed a direct connection to this subject, whether in the title, keywords, or abstract. Book reviews, magazine articles, and publications without apparent relationship to the topic were excluded. We put extra emphasis on those articles which not only mentioned hackathons but also included insights on how to best execute them.
- Time: A preliminary search of hackathon literature revealed that the earliest mention of the term in an article’s title, keywords, or abstract was in 2007, a similar result to previous reviews of hackathons (e.g., Olesen & Halskov, 2020). We, therefore, included articles published between 2007 and 2022.
- Research with vs. on hackathons: in their review of the state of academic research on hackathons, Olesen and Halskov (2020) differentiated two types of research: research with hackathons and research on hackathons. Articles that fall under research with hackathons use hackathons as part of the research approach itself rather than being the object of the study. Conversely, research on hackathons describes articles that study the hackathon format to contribute to our understanding of the phenomena itself. In this review, we were interested in both types of research, given that both can offer insights and produce guidelines on how to best conduct a hackathon.

### Search strategy

**Fig. 1:**
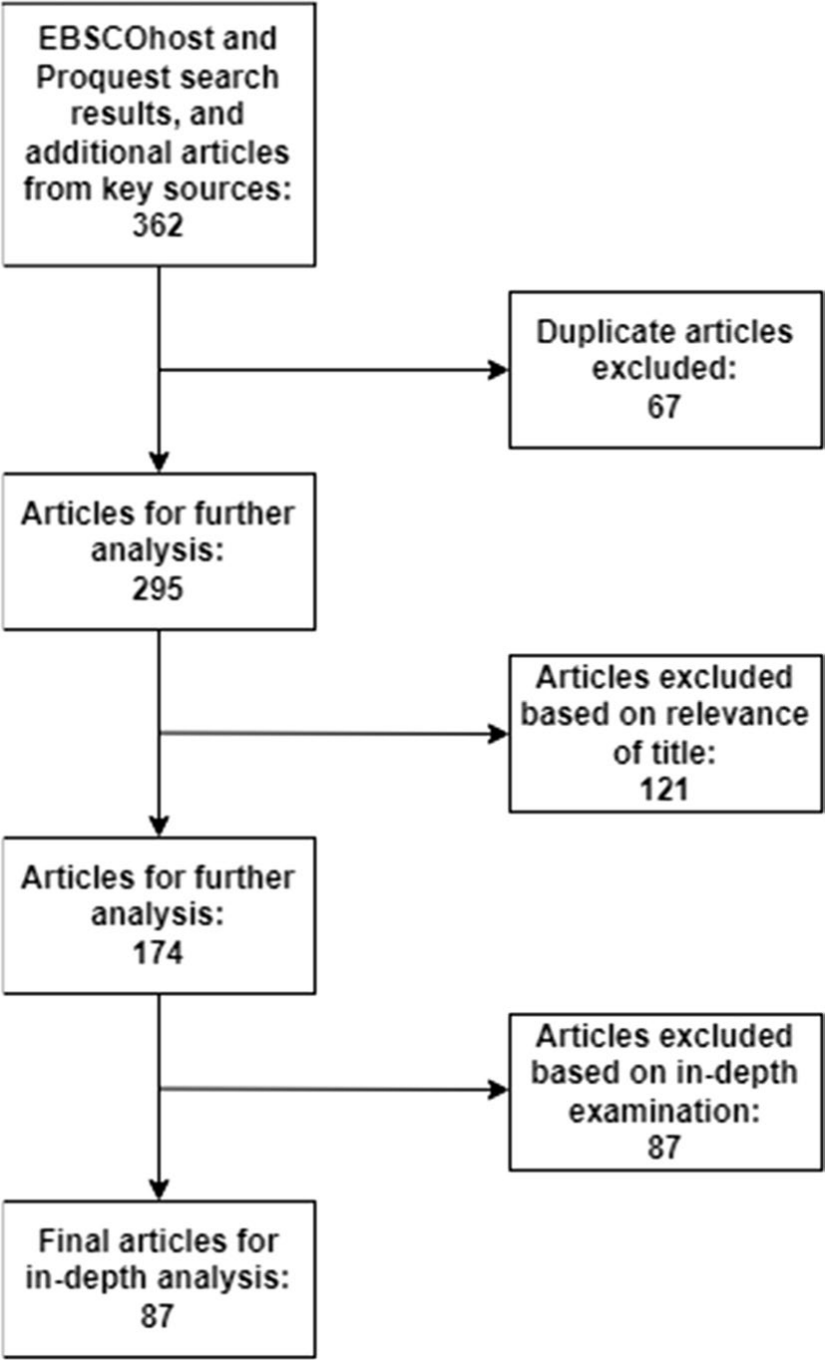
Literature review electronic search iterations

The electronic search strategy incorporated the string “hackathon*” using the Proquest and EBSCOHost databases. Appropriate synonyms, misspellings, and truncations were included. With these terms, we searched the databases in the following categories: title, keywords, and abstract. As per our inclusion/exclusion criteria, we limited our search to articles released between 2007 and 2022. The keywords search resulted in 215 hits for the Proquest database and 110 hits for the EBSCOHost database. In addition, searching for the keyword “hackathon*” (after 2007) produced 16,300 results on Google Scholar. These results were primarily used as a supplementary database, providing specific articles used depending on their impact (citations) and their potential for broadening the discussion. Additional existing reviews relevant to the broader subject area were consulted to provide a context for the studies identified for the current review. This process gave us an initial count of 362 articles (as shown in Fig. 1). Eliminating duplicates (articles that appeared in multiple keyword searches) further reduced the set to 295. The penultimate step was to exclude articles based on the apparent irrelevance of their title to the subject matter, which brought our search to 174. Finally, complete copies of these 171 papers were read and closely examined to determine whether they met all of the above inclusion criteria, bringing our set to its final hit count of 87.

## Classifications of hackathons

### Hackathon focus

As a first step, organizers must decide on the focus or orientation of the hackathon. Scholars describe tech-oriented vs. issue-oriented hackathons (Briscoe, 2014; Lodato & DiSalvo, 2016). Tech companies usually host tech-oriented hackathons, mainly appealing to software developers, application creators, product designers, etc. Technological hackathons are also held in education, especially in computer science or engineering faculties, where students develop their technical abilities and learn to create technological products (Byrne et al., 2016). In medicine, hackathon participants develop innovative medical technologies to improve patients’ well-being (Wang et al., 2018b).

A significant advantage of a tech-oriented hackathon is the capacity to find innovative and tangible solutions quickly. Another advantage is the inherent adaptation and compatibility with other tech-related innovative methodologies, such as agile development (Abrahamsson et al., 2017) and the “Lean Startup” (Blank, 2013), focusing on customer needs. In other words, tech-oriented hackathons enable organizers to achieve tangible and action-ready results that have been updated according to market needs quickly and cost-effectively.

At the same time, there are two disadvantages to these events. First, finding and recruiting participants with the appropriate professional background is often difficult. Second, such events may be costly and challenging to organize, requiring advanced technological tools (Briscoe, 2014). Third, most technologies cannot be developed quickly, which can impair the quality of solutions and, consequently, their implementation. Finally, tech-oriented hackathons may suffer from an over-focus on technological development rather than on aspects that support the product, such as user experience, a fitting business model, etc.

In contrast, issue-oriented hackathon events aim to solve broader business or social problems (Briscoe, 2014). These appeal to a wider range of participants from all walks of life and often contain more diverse teams from different disciplines. Issue-oriented events typically include between 30 and 50% non-tech participants compared to about 10 to 20% in tech-centric events. Issue-oriented hackathons take place in areas such as academia (Maaravi, 2020), governments (Johnson & Robinson, 2014), businesses (Pe-Than et al., 2018), and more. The significant advantage of issue-oriented over tech-oriented hackathons is the capacity to build heterogeneous teams with diverse professional experience. There is ample evidence that such teams exhibit greater creativity than homogeneous teams (Groen & Calderhead, 2015). In addition, issue-oriented hackathons produce a wider range of products, depending on the organizers’ challenge.

Nevertheless, teams without deep technological experience have difficulty creating tangible tech products during the hackathon. Therefore, organizers must ensure that all teams include tech-savvy participants (Briscoe, 2014). In addition, issue-oriented hackathons require diverse players’ joint work and expertise, complicating the realization of its products after the event (Angarita & Nolte, 2020).

### Hackathon design choices

After selecting the event focus, organizers need to decide on the characteristics of their hackathon (Pe-Than et al., 2018). The different characteristics are (1) Selective vs. open hackathon recruitment, (2) Competitive vs. collaborative hackathon atmosphere, and (3) Physical vs. virtual vs. hybrid hackathons. It is important to emphasize that the organizer is free to combine all these characteristics into the most effective hackathon design according to their objectives.

#### Selective vs. open hackathon recruitment

Nolte et al. (2020) characterized two approaches for recruiting hackathon participants—selective and open recruitment. Open recruitment is focused on obtaining as wide a range of participants from various fields and industries as possible to encourage interdisciplinary connections and fertilization. The advantage is that such diversity has been shown to promote good results throughout the hackathon and after its end (Groen & Calderhead, 2015; Paganini & Gama, 2020). Another advantage is participants’ higher motivation due to their free choice to participate (Nolte et al., 2018).

The open recruitment approach is appropriate when goals include building a community around a particular subject (Nolte et al., 2020). It also allows organizations to identify talents and recruit motivated and skillful employees (Herala et al., 2019). In addition, the organization will benefit from positive public relations as an innovative and open organization (Nolte et al., 2018). On the other hand, the open recruitment approach allows for little control over the choice of participants. Another related disadvantage is exposing sensitive inside information to external players (Herala et al., 2019).

On the other hand, the selective recruitment approach entails organizers inviting participants who meet specific knowledge requirements, are related to the organization or are part of a content-specific community (Huppenkothen et al., 2018). Organizations use this approach to encourage and promote a culture of innovation or generate and strengthen social ties. Examples include hackathons intended for employees of a particular organization, students in a specific faculty, professionals relevant to the hackathon theme, or a particular demographic group. This approach allows engineering a concrete and accurate result by selecting participants who can accurately contribute to or benefit from the hackathon event. Moreover, it is possible to ensure in advance that each group participating in the hackathon will have an ideal mix of skills and knowledge to achieve the best results for successful long-term projects. In addition, closed hackathons are suitable for organizations required to maintain confidentiality. Finally, participants often come from the same professional culture with the same norms, allowing for smoother teamwork and coordination (Nolte et al., 2020).

#### Competitive vs. collaborative hackathon atmosphere

De Winne et al. (2020) distinguished between competitive events—in which the primary goal of the participants is to win prizes and benefits—and those of a collaborative nature—in which participants cooperate to achieve a shared goal. Hackathons are usually structured such that teams’ products and ideas are judged by experts, and the teams compete for prizes. The competitive atmosphere increases external motivation (Maaravi, 2020) and pushes teams to offer unique ideas (Nolte et al., 2020). Nevertheless, researchers have found that a competitive atmosphere may lower intrinsic motivation (Cwikel & Simhi, 2021). On the other hand, creating a supportive and less competitive atmosphere may promote productive creativity, increase performance, and increase participant satisfaction (Calco & Veeck, 2015; Cobham et al., 2017; Fadlelmola et al., 2021). A possible solution is to offer several small prizes instead of “the winner takes it all” approach (De Winne et al., 2020). Research has also pointed to women’s preference for a collaborative atmosphere, manifested in low participation rates of women in competitive hackathons (Paganini & Gama, 2020), which undermines the unique contribution of gender diversity to group performance (Woolley et al., 2010). Another study examined the effects of a collaborative atmosphere by analyzing a hackathon executed as part of a marketing course. The study found that the hackathon encouraged creativity, critical thinking, and innovation thanks to the organizers’ emphasis on a more relaxed and creative atmosphere. This was partly achieved since the hackathon only accounted for 10% of the final course grade, which reduced stress and contributed to a positive and educating experience (Calco & Veeck, 2015).

Competitive hackathons reduce inter-team (as opposed to intra-team) communication. Therefore, they are less suitable when the primary purposes are to foster collaboration and networking or to promote a solution for a common problem. Thus, collaborative hackathons are particularly suited to advancing a social goal, in which teams collaborate to reach a solution (Möller et al., 2014; Panchapakesan et al., 2019). Examples of this type of hackathon can be found in those designed to solve urban and social problems or those run by non-profit organizations. Hackathons that teach a new tool or subject will also benefit from a collaborative approach (Nolte et al., 2020). Two examples of collaborative-style hackathon techniques include (1) “unconference” sessions (Stoltzfus et al., 2017), in which participants take a break from their current team and pitch their ideas to members of other teams for feedback; and (2) team switching (Porter et al., 2017), in which participants switch team at regular intervals to allow for diversity and the possibility of meeting new members.

Nevertheless, organizers should be wary of potential mismatches between the hackathon’s competitive nature and the participants’ personal goals. For example, Kos (2019) details a competitive hackathon in which participants had non-competitive goals as evidenced by their behavior: some individuals focused on exploration, meaning they used the learning opportunities and time-constrained formats for non-hackathon-related objectives; some preferred dabbling, meaning that they sought to learn and explore a wide array of hackathon-related topics and not necessarily their projects; and yet others favored observing, meaning that they did not partake in many of the activities throughout the hackathon yet enjoyed merely being present. Thus, organizers should strive to include opportunities for participants with non-competitive goals and include participatory activities aside from competition (Kos, 2019).

#### Physical vs. virtual vs. hybrid space hackathons

Traditionally, hackathons are conducted in a designated physical space, encouraging communication between participants, increasing participants’ engagement, and adding to the hackathon’s sense of importance (Teasley et al., 2000). However, there are some disadvantages to physical space hackathons. First, it is costly as it is necessary to provide participants with adequate food, working conditions, and sometimes accommodation (Briscoe, 2014). Second, it may reduce the size and diversity of the participant pool since it requires potential participants to live or work close to the event location. Third, the traditional atmosphere in physical hackathons may exclude various populations, such as women or underrepresented ethnic groups (Paganini & Gama, 2020).

The outbreak of the coronavirus (COVID-19) pandemic in 2019 (Singhal, 2020) and the subsequent need for social remoteness led to a significant reduction of physical space hackathons. Government constraints, coupled with people’s fears of being in a crowded and contagious environment, have led to a sharp rise in virtual hackathons (Bertello et al., 2022; Ramadi & Nguyen, 2021; Wang et al., 2021). Virtual hackathons are conducted using remote communication software such as Zoom (Bolton et al., 2021) or a “mashup” of several applications (Maaravi & Heller, 2021) and may have several advantages. First, virtual hackathons may contribute to participant heterogeneity. Numerous studies have argued that gender- and skillset-diverse groups can achieve better results in hackathons (e.g., Paganini & Gama, 2020; Wang et al., 2018b). For example, research findings have shown that gender diversity led to more radical innovation within R&D teams (Díaz-García et al., 2013). Additionally, skillset diversity may promote the cross-pollination of ideas from a wide range of expertise (DePasse et al., 2014). Second, virtual hackathons enable great geographical and cultural diversity, which creates an extensive network of connections across cities, countries, and even continents (Bertello et al., 2022; Bolton et al., 2021). Similarly, the virtual hackathon lowers the barriers preventing underrepresented populations (women, specific ethnic groups, etc.) from participating in physical hackathons. For example, one of the main barriers that limit women’s participation in hackathons is childcare, which virtual hackathons solve by allowing participants to participate from their homes (Hardin, 2021). Third, virtual hackathons can quickly react to and address unexpected crises that require innovative solutions (e.g., the COVID-19 crisis) by involving the most relevant parties regardless of physical limitations (Bertello et al., 2022; Franco et al., 2021). Finally, virtual hackathons reduce logistical needs, such as the venue for the event, food, and seating arrangements, thus lowering financial expenses (Bertello et al., 2022; Bolton et al., 2021).

At the same time, there are potential drawbacks to virtual hackathons. One is the difficulty of locating the source of the ideas (Bolton et al., 2021), which involves credit and originality issues. Second, virtual hackathons limit the ability to form close and meaningful relationships, a prominent motivation for hackathon participation (Kolog et al., 2016; Maaravi, 2018). Research has shown that both the quality and the number of connections in the virtual space are inferior to those formed in physical events (Thellman et al., 2016). One reason is that teams are often divided into virtual rooms with few participants. Finally, virtual hackathons might distance less tech-savvy participants due to the need to manage multiple online platforms simultaneously (Bertello et al., 2022). This can be overcome by providing participants with a concise guide on navigating the specific virtual tools and designating which tool will be used for which purpose.

A possible solution to the limitations of both physical- and virtual-space hackathons is to use a hybrid design. In hybrid hackathons, some stages are executed in person and some online (Khan et al., 2021; Mubaraz et al., 2021; Porras et al., 2021). Alternatively, some participants participate in the physical space while others join virtually (Ribault et al., 2022). Research has shown that hybrid hackathons show comparable results both on participant-oriented variables of interest (such as attendance, quality of participation, cooperation, leadership, etc. See: Mubaraz et al., 2021), and project-oriented variables of interest (such as ideation, idea selection, information gathering, and perceived quality of the final product. See: Khan et al., 2021; Porras et al., 2021). Thus, a hybrid hackathon format allows organizers to capitalize on the most important aspects of physical and virtual hackathons (Porras et al., 2021). For example, when establishing and promoting a team spirit is essential (e.g., team formation stage), organizers can decide on conducting them in a physical space, where socialization processes are most effective (Porras et al., 2021).

## Guidelines for successful hackathons

**Fig. 2:**
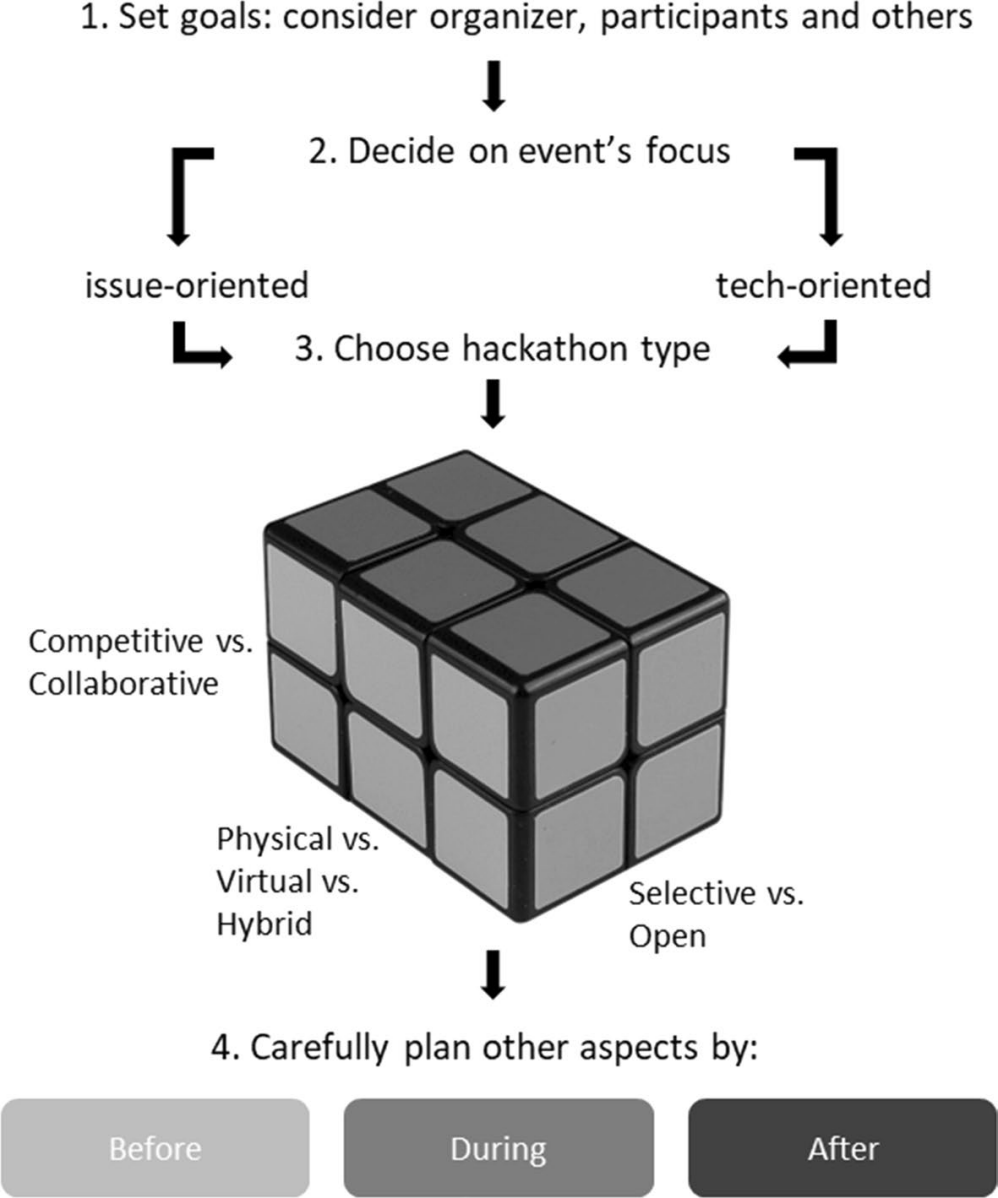
Major steps in planning a hackathon

This section is divided into three parts according to the chronology of hackathons events: best practices and research-based recommendations regarding procedures, arrangements, and activities before, during, and after the event. The chronology of these steps is illustrated in Fig. 2.

### Before the event

#### Defining the objectives of the hackathon

While there are at least two key players in most hackathons—organizers and participants—many more players are typically involved: mentors, sponsors, judges, etc. Since each player has distinct interests and motivations (DeFranco et al., 2021), it is essential to consider all stakeholders’ goals and needs (Briscoe, 2014). For example, academic hackathons aim to increase participants’ skills and intrinsic motivation by learning from practical experience (Aungst, 2015; Chandrasekaran et al., 2018; Maaravi, 2018, 2020). In contrast, in urban and public sector hackathons, goals typically include increasing citizen participation in decision-making (Johnson & Robinson, 2014) or improving organizers’ perceptions as innovative and attentive to citizens’ needs (Herala et al., 2019). Medical or scientific hackathons usually include the invention of life-saving medical developments (e.g., finding unique technological solutions for people with dementia; Trainer et al., 2016), creating applications to improve public health systems, or raising awareness of critical medical issues (e.g., early detection of diseases, improving the medical condition in developing countries; Wang et al., 2018b).

In the business domain, a central goal for organizers is to enhance innovation processes, whether directly by creating new products or indirectly by increasing employees’ motivation for innovation (Herala et al., 2019). Occasionally, organizations seek to realize other goals, such as identifying talented employees for future promotion or recruiting quality personnel (Zionia & Sathyapriya, 2021). Other purposes are to improve teamwork, unite employees, and foster an overall positive atmosphere of action and fruitful cooperation. Moreover, as in the public sector, hackathons potentially create positive public relations, thus branding organizers as innovative, social, creative, and ambitious companies (Nolte et al., 2018). Research has also investigated participants’ goals and found that they primarily include learning new knowledge or practicing existing skills (Kolog et al., 2016). Second, participants use hackathons to deepen their professional network (Cardwell et al., 2021; Gabrilove et al., 2018; Wang et al., 2018b) and create communities around shared goals (Bertello et al., 2022; Cardwell et al., 2020; McLeod et al., 2019). Third, some hackathon participants attend them to enhance their personal or social skills such as teamwork, leadership, and project management (Cwikel & Simhi, 2021; Groen & Calderhead, 2015; Hogan & Young, 2021). Finally, others have more material goals, such as winning prizes, receiving funds, and meeting potential partners to promote their projects or ventures (Nolte et al., 2020).

All goals should be precise, achievable, and realistic. A mismatch between the desired goals of those involved can cause disappointment among participants and organizers, impair the innovation process, and provoke negative emotions (Bell et al., 2019; Cobham et al., 2017; Pihlajamaa & Merisalo, 2021).

#### Defining hackathon design and focus

After setting the goals, organizers need to decide on the event’s focus, followed by the design of the hackathon—an open or selective recruitment method, competition or collaboration hackathon, and a physical, virtual, or hybrid space hackathon. See Sect. “Hackathon design choices” for a more in-depth explanation of the different design characteristics and Fig. 2 for an overview.

#### Participants and team building

The following step is to decide on the identity of the participants. Organizers should consider the participants’ abilities and goals (Pirker et al., 2018) and adjust the objectives accordingly (Cobham et al., 2017). Participants’ identities are often contextually predetermined—for example, students taking the course (Groen & Calderhead, 2015) or company employees (Briscoe, 2014). In other cases, organizers must decide who the desired participants are regarding their skill set, experience, demographics, and more. Then, organizers must reach out to potential participants by marketing the event using the necessary media tools. Setting up a unique website or landing page for the event is recommended to allow participants to register and upload their experience and skillset information. These marketing channels should present factual information and incentives to convince participants to participate in the event. Then, it is crucial to review participants’ resumes, filter out irrelevant candidates, and move on with the appropriate ones (Nolte et al., 2020).

Simultaneously, organizers should decide on the nature and structure of the teams (Safarova et al., 2015; Trainer et al., 2016). Organizers can allow pre-registered teams to come together to the event, regardless of the team’s diversity, which may improve teamwork yet jeopardize creativity, as such teams will usually consist of members from the same field (Nolte et al., 2020). Conversely, organizers can build the teams themselves to ensure they are more professionally diverse (Bell et al., 2019). As previously stated, studies have indicated that diverse teams lead to more creative ideas than homogenous teams. Such teams are also more efficient and can work on multiple tasks simultaneously (Bertello et al., 2022; Kurtzberg, 2005; Pathanasethpong et al., 2017; Tadmor et al., 2012). Notably, some participants may have more significant hackathon expertise (i.e., experience in participating in hackathons) than others. Matching more experienced participants with those with less experience has been hypothesized to contribute to overall team success (Flus & Hurst, 2021).

Also, it is recommended that team size be limited to three to sex individuals so that each member will have more influence, a more significant learning experience, and increased identification with the team (Day et al., 2017; Herala et al., 2019; Maaravi, 2018). It is recommended to inform participants about team composition in advance to allow them to prepare accordingly. The advantage of announcing the teams in advance is that they can get to know each other, understand their strengths and weaknesses, and start developing ideas and thinking processes before the event (Horton et al., 2018; Nandi & Mandernach, 2016).

#### Announcing the hackathon’s challenge and preliminary meeting

The next issue to consider is presenting the hackathon challenge. Past studies have shown a clear relationship between an early and clear presentation of the hackathon challenge and both the success of the hackathon for organizers and participants’ satisfaction (Fadlelmola et al., 2021; Kitsios & Kamariotou, 2019; Nolte et al., 2018; Trainer et al., 2016). Such preliminary info sessions are usually held as a meeting or webinar and help filter out irrelevant participants, facilitate participant preparation, and create greater efficiency in teamwork during the event (Calco & Veeck, 2015; Kopeć et al., 2018). Additionally, a study of online hackathons conducted during the COVID-19 pandemic emphasized the importance of adopting a broad scope when facing grand (large-scale, global) challenges (Bertello et al., 2022). By defining the hackathon’s challenge in general and broader terms (instead of overly specific and narrow), organizers can: (a) encourage disruptive rather than incremental innovation and (b) stimulate different perspectives and promote curiosity by allowing some ambiguity in the challenge’s definition. Based on these findings, we recommend that organizers present the challenge early during the hackathon and do so broadly.

#### Logistics and rules

Logistically, it is vital to choose a proper location for the event and ensure its appearance is fitting and motivating. Organizers should ensure that the venue is open and available throughout the event without any external interruptions. It is also essential to take care of constant food breaks, flexible seating arrangements, internet availability, whiteboards, notebooks, writing utensils, and sufficient team space. Also, there is a preference for large rooms or halls with windows and ventilation (Briscoe, 2014). If a global participant pool is aimed, lodgings should be booked and adequately marketed to encourage participant enrollment (Hackseq Organizing Committee, 2017). Although to a lesser degree, virtual hackathons also have logistic nuances: preparing dedicated online platforms (Maaravi & Heller, 2021), providing support to teams both technically and in their creative process, and creating an atmosphere of inspiration and innovation despite the physical distance (Bolton et al., 2021).

The organizer must define the rules for the event in advance and communicate them to all players. Rules should be set regarding the event schedule, stages, and judging phases (Trainer et al., 2016). Additionally, rules regarding the culture of discussion and behavior should be defined, such as participants’ ethics and copyright rules regarding hackathon products, mainly to avoid legal incidents in post-hackathon project implementation (Herala et al., 2019).

#### Competition, judges, and mentors

When planning the hackathon’s competitive aspect, organizers should consider what was previously reviewed in Sect. "Competitive vs. collaborative hackathon atmosphere": a very competitive atmosphere with high-value prizes creates extrinsic motivation to stand out, but an atmosphere of collaboration leads to improved creativity, better teamwork, and intrinsic motivation. As part of these preparations, organizers are advised to decide on the rewards, incentives, presenting the ideas to the judges, and how winners are selected (e.g., judge panel vs. popular vote). Since the quality of the prizes affects participants’ extrinsic motivation (Ferreira & Farias, 2018; Maaravi, 2018), organizers will occasionally have to find sponsors for the hackathon’s prizes (Nolte et al., 2020). Besides their financial support, highly prestigious and sought-after sponsors can significantly influence people’s motivation to participate and succeed (Franco et al., 2021).

The identity of the judges varies: they can be external judges—such as experts in the field, senior scholars, community leaders, or representatives from various companies—or internal judges such as organization managers and leaders (Major League Hacking, 2019). Generally speaking, it is recommended to attract high-profile external judges with the necessary knowledge and expertise (Kitsios & Kamariotou, 2019), which teams try to impress to advance their careers and professional pursuits (Nolte et al., 2020).

A final issue is the use of mentors throughout the event. Like judges, the organizers should determine their identity, recruit them in advance, and coordinate expectations (Kolog et al., 2016). Mentors can guide specific teams or rotate between them. As with judges and sponsors, recruiting high-profile mentors can increase participants’ motivation and satisfaction (Lara & Lockwood, 2016) by improving learning processes, expanding participants’ networks, and exposing them to executives capable of contributing to their careers (Day et al., 2017; Kitsios & Kamariotou, 2019; Nolte et al., 2020). Notably, the mentor pool should comprise diverse profiles and skills to best interact with the mentees’ competencies and maximize outcomes (Franco et al., 2021; Pathanasethpong et al., 2017).

### During the event

Once all preliminary planning has been completed, organizers should form an operations team to produce the event and address the ongoing issues. Alongside operations, this team will help the organizers understand participants’ progress, connect teams with mentors, aid with tech problems, and alert the teams about schedules.

#### Greetings and kickoff

Since one of the primary goals of participants is socializing and networking (Wang et al., 2018b), organizers should allow enough time for social interactions before the kickoff (Angelidis et al., 2016; Aryana et al., 2019). These interactions are crucial to creating a friendly and embracing atmosphere that reduces interpersonal barriers and facilitates cooperation (Panchapakesan et al., 2019). Following this initial social networking phase, participants should be gathered in one place for introductions, announcements, instructions, and any other guidelines and clarifications regarding the hackathon. Organizers should present the topics of the event, the expectations from the participants, the goals they want to achieve, the prizes and method of judgment, define the timetable, and go over the basic rules and emphases. Finally, this is also the time to present the mentors and judges, including their professional experience, and provide information on the operational team contact list (Karlsen & Løvlie, 2017; Purwanto et al., 2019; Rosell et al., 2014).

#### Temporal structuring and coordination

Previous studies in organizational settings with traditional time structures have found that time pressure has detrimental effects on innovation and creativity (Amabile et al., 2002; Perlow, 1999; Perlow et al., 2002). On the other hand, hackathons are unique because their time frame is limited by design. In this regard, Lifshitz-Assaf et al. (2021) examined the temporal structures of 13 projects developed in assistive technology hackathons. Seven of these projects decided to import temporal structures from established organizational innovation processes and adjusted them to fit into the hackathon time frame. In other words, the teams worked in complete coordination, which previous research in traditional organizational settings has found to be conducive to the innovation process by enabling individuals to cooperate and reach decisions quickly (Ben-Menahem et al., 2016; Malone & Crowston, 1994; Steinhardt & Jackson, 2014). Nevertheless, Lifshitz-Assaf et al. (2021) found that all these teams failed to produce a working product by the end of the hackathon. They attributed this result to the accelerated innovation conditions, which rendered teams using full coordination unable to adapt to the high ambiguity in what is coined the “speed trap” (Perlow et al., 2002). By contrast, the six teams that succeeded in producing a working product were marked by their perception of the hackathon as a wholly distinct innovational process. Thus, instead of compressing traditional temporal structures, they used novel structures characterized by adaptive coordination processes. In adaptive coordination processes, participants start with a minimal basis for coordination and increase it as their work progresses while sensing and adjusting to their teammates’ work. This approach enabled the needed flexibility to adapt to ambiguous circumstances and led to their ultimate success, even in the face of redundancies and mistakes. Thus, hackathon organizers should preemptively recommend adaptive coordination processes to the participants.

#### Ideation, idea selection, and execution

As activity begins, teams should be encouraged to dedicate enough time for discussion, including brainstorming, and divergent thinking activities, which have proven to be effective in ideation processes (Maaravi et al., 2020). One possibility that emerges from the literature on creativity is to appoint a mentor as an ideation facilitator. Wilson (2013) described some critical behaviors of good brainstorming facilitators, including preventing participants from offering premature criticism, encouraging the flow of ideas, focusing on quantity rather than the quality of ideas, and promoting tolerance for radical ideas. During the idea selection phase, it is advised to have organizers or mentors approve preliminary ideas before their execution to make sure they align with the hackathon’s goals (Angelidis et al., 2016; Birbeck et al., 2017).

Once teams select their leading idea and start working on its execution, organizers and mentors should monitor their progress to ensure all team members are engaged (Birbeck et al., 2017; Soltani et al., 2014).

#### Judgement

At the end of the production phase, the judges typically review the different products or ideas, ask questions, express their opinions and choose the winners. Winners can be selected by a jury or based on a popular vote. Experts are better suited as judges if the judging criteria are complex since they can determine if an idea or prototype is feasible and meets the challenge (Boisen et al., 2017; Suominen et al., 2018; Wilson et al., 2019). On the other hand, a popular vote may be more appropriate if the desired result is a solution for a common and well-known problem. Some standard judgment criteria include market appeal, creativity, originality, completeness, and difficulty level.

### After the event

The announcement of the winning teams signifies the end of the event but not the completion of the hackathon. Research points to several activities that organizers of successful hackathons perform. First, they follow up with all involved players several days after the event through surveys and personal interviews (Nolte et al., 2018). Such data may help improve future performance and locate promising talents or projects that did not win the competition. Second, organizers should stay in touch with leading teams to further assist and support the development of their products and facilitate communication with key people in the organizations relevant to the hackathon’s challenge (Groen & Calderhead, 2015; Herala et al., 2019). Finally, organizers should encourage direct conversation between the participants themselves and between them and organizations’ representatives by creating contact boards, forums, and other social network platforms (e.g., Facebook or Linkedin groups). Such open and direct communication may encourage novel ideas and continued work on those developed during the hackathon (Trainer et al., 2016).

### Factors affecting post-hackathon continuity

Scholars have suggested multiple factors that may affect the continuity of hackathon projects and products after the event ends. First, ideas that contribute to participants’ career development or offer meaningful learning experiences have a higher chance of continuing (Nolte et al., 2018, 2020; Pe-Than et al., 2022). Similarly, one study found that teams with a career-oriented leader showed increased project continuity and success (Pe-Than et al., 2022). Second, research has found that projects that received support from the organization, an orderly place to work within the organization, and the coupling of additional professionals and mentors for professional help, were likely to continue with their hackathon projects (Nolte et al., 2018, 2020). Third, projects that are essentially follow-up products or are new features within an existing product have a higher chance of successful continuity than entirely new products (Nolte et al., 2018, 2020; Pe-Than et al., 2022). Fourth, offering post-event incentives, such as additional rewards, may increase the chances of project continuity (Wang et al., 2018b). Finally, Stoltzfus et al. (2017) suggested encouraging teams to participate in a follow-up program and designating an organizational champion with sufficient time to orchestrate these efforts (Hecht et al., 2014).

## Conclusions

**Fig. 3:**
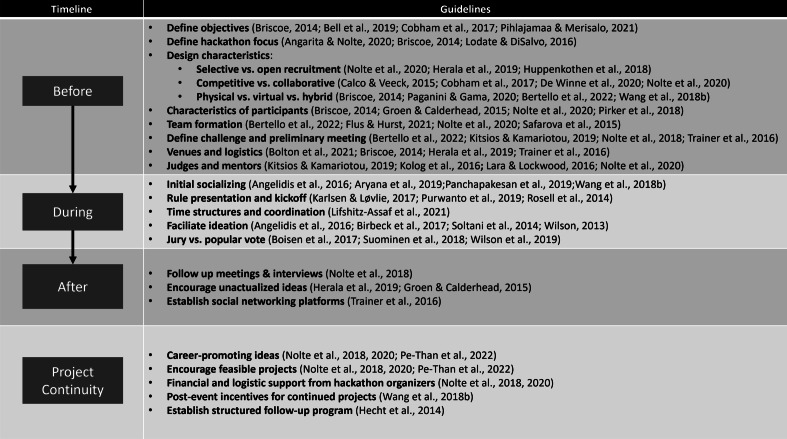
Summary of guidelines for running successful hackathons. For brevity and clarity, only the four most essential references were included for each guideline. The rest can be found in their respective sections

The innovation imperative characterizes our current era: organizations must innovate to remain relevant in the competitive markets and face novel business, social and environmental challenges. This innovation imperative has pushed scholars and organizations alike to search for methods to promote innovation. One example is the open innovation approach, which seeks innovation from various sources and is not limited to internal organizational processes (Chesbrough, 2006). While the open innovation approach is more strategic, hackathons are among the most common innovation techniques (Maaravi, 2020) and can be part of a broader open innovation strategy or not. The current article introduces the hackathon phenomenon by reviewing the relevant academic and professional literature. Our thorough literature review has generated guidelines, recommendations, and tips on successfully producing a hackathon event, summarized in a chronological chart (see Fig. 3).

Before the event, it is essential to understand the hackathon’s goals and design its attributes accordingly. The main attributes include focus (tech-oriented / issue-oriented) and design characteristics: recruitment method (selective/open), atmosphere (competition/collaboration), and location (physical/virtual/hybrid). These affect the scheduling, prizes, participant pool, and overall execution of the hackathon. It is crucial to coordinate expectations with participants regarding the rules and objectives of the hackathon, and organizers should recruit the appropriate judges, sponsors, and mentors for the topic and participants.

During the event, an operations team should ensure the event’s smooth execution and support the participants, judges, and mentors. Organizers should allow enough time for networking and social interaction at the beginning of the event and then present the rules, judges, judging criteria, and prizes. During teamwork stages, mentors should act as facilitators to team ideation, encouraging participants to offer multiple ideas and making sure each voice is heard. Besides answering the hackathon’s challenge, ideas that also drive participants’ careers and promote their learning and growth are more likely to come to fruition.

After the hackathon, organizers and organizational innovation champions should follow up with participants using questionnaires, interviews, and even team meetings to understand their attitudes and feedback. In addition, using a social network platform for the participants to communicate after the event will help the continuity of the hackathon’s products. Finally, post-event support for feasible ideas in the form of awards, infrastructure, and professional advice will also increase the likelihood that ideas will materialize.

The current article contributes to the literature by providing an in-depth, step-by-step guide on best executing a hackathon. Its similarities and differences with related works can be found in Table 1.

### Limitations and future research

However, our article and the conclusions that can be drawn from it have several limitations. First, since hackathons are a 21st-century phenomenon and have only gained academic traction in recent years (Olesen & Halskov, 2020), more in-depth research is needed to expand the knowledge around the subtleties of this intriguing phenomenon. For example, virtual hackathons, which saw a significant surge in usage during the COVID-19 pandemic, were seldom investigated (Maaravi & Heller, 2021). Another example is the relationships between hackathon organizers (e.g., academia) and the companies sponsoring the challenges. Finally, internal organizational decision-making processes that lead to selecting specific subjects for the hackathons are also interesting and relevant for innovation research.

Additionally, existing studies are limited to mainly correlative case studies, which do not allow for a proper understanding of the causal processes underlying effective hackathon execution. Experimental research paradigms should deepen our understanding of the hackathon phenomenon. For example, studies can examine participant-, organization-, or event-level variables of interest by comparing the outcomes of two hackathons that are identical in all characteristics except one (e.g., staff diversity, judges’ identity, virtual versus physical versus hybrid, etc.).

Finally, our guidelines are based on a literature review without statistical methods to examine past hackathon results and outcomes. This was mainly done due to the research methodology used in past research (i.e., case studies). Future research could overcome this limitation by using advanced qualitative (such as collecting previous hackathons’ post-event surveys and interviews) and quantitative methods (such as meta-analysis) to offer data-driven conclusions on how to best plan and execute hackathons.

### Conclusion

In conclusion, the present article gives scholars, managers, and innovation champions in organizations guidelines on producing a successful hackathon based on the most up-to-date academic and professional knowledge. This article summarizes the literature on this new and promising research topic and provides a starting point for further exploration to deepen our understanding of hackathons.

## Data Availability

Not applicable.

## Abbreviations

R&D: Research and development
COVID-19: Coronavirus disease 2019
